# Change in diaphragm and intercostal muscle thickness in mechanically ventilated patients: a prospective observational ultrasonography study

**DOI:** 10.1186/s40560-019-0410-4

**Published:** 2019-12-02

**Authors:** Nobuto Nakanishi, Jun Oto, Yoshitoyo Ueno, Emiko Nakataki, Taiga Itagaki, Masaji Nishimura

**Affiliations:** 1 Emergency and Critical Care Medicine, Tokushima University Hospital, 2-50-1 Kuramoto, Tokushima, 770-8503 Japan; 2 Tokushima Prefectural Central Hospital, 1-10-3 Kuramoto, Tokushima, 770-8539 Japan

**Keywords:** Diaphragm, Intercostal muscle, Atrophy, Ultrasonography

## Abstract

**Background:**

Diaphragm atrophy is observed in mechanically ventilated patients. However, the atrophy is not investigated in other respiratory muscles. Therefore, we conducted a two-center prospective observational study to evaluate changes in diaphragm and intercostal muscle thickness in mechanically ventilated patients.

**Methods:**

Consecutive adult patients who were expected to be mechanically ventilated longer than 48 h in the ICU were enrolled. Diaphragm and intercostal muscle thickness were measured on days 1, 3, 5, and 7 with ultrasonography. The primary outcome was the direction of change in muscle thickness, and the secondary outcomes were the relationship of changes in muscle thickness with patient characteristics.

**Results:**

Eighty patients (54 males and 26 females; mean age, 68 ± 14 years) were enrolled. Diaphragm muscle thickness decreased, increased, and remained unchanged in 50 (63%), 15 (19%), and 15 (19%) patients, respectively. Intercostal muscle thickness decreased, increased, and remained unchanged in 48 (60%), 15 (19%), and 17 (21%) patients, respectively. Decreased diaphragm or intercostal muscle thickness was associated with prolonged mechanical ventilation (median difference (MD), 3 days; 95% CI (confidence interval), 1–7 and MD, 3 days; 95% CI, 1–7, respectively) and length of ICU stay (MD, 3 days; 95% CI, 1–7 and MD, 3 days; 95% CI, 1–7, respectively) compared with the unchanged group. After adjusting for sex, age, and APACHE II score, they were still associated with prolonged mechanical ventilation (hazard ratio (HR), 4.19; 95% CI, 2.14–7.93 and HR, 2.87; 95% CI, 1.53–5.21, respectively) and length of ICU stay (HR, 3.44; 95% CI, 1.77–6.45 and HR, 2.58; 95% CI, 1.39–4.63, respectively) compared with the unchanged group.

**Conclusions:**

Decreased diaphragm and intercostal muscle thickness were frequently seen in patients under mechanical ventilation. They were associated with prolonged mechanical ventilation and length of ICU stay.

**Trial registration:**

UMIN000031316. Registered on 15 February 2018

## Background

Critically ill patients experience atrophy of various muscles including respiratory muscles, with diaphragm muscle atrophy garnering increasing attention. Patients undergoing prolonged mechanical ventilation experience sustained diaphragm muscle loss, which leads to worse clinical outcomes [1].

Mechanical ventilation can affect not only the diaphragm but also the intercostal muscles, as demonstrated in animal studies [2]. Although diaphragm is the main respiratory muscle, several other respiratory muscles contribute to the respiratory effort as well. Specifically, when respiratory workload increases, other respiratory muscles are more active than the diaphragm [3]. Among these, intercostal muscles are one of the most important respiratory muscles in manipulating the movement of the rib cage [4].

Although diaphragm atrophy was reported to affect clinical outcomes in mechanical ventilation [1, 5], changes in other respiratory muscles during mechanical ventilation are not well investigated. Since diaphragm and intercostal muscles play an important role in critically ill patients, it is reasonable to hypothesize that the thickness of both diaphragm and intercostal muscles could change over the mechanical ventilation. Therefore, we evaluated changes in diaphragm and intercostal muscle thickness and the potential relationship with patient characteristics, medications, ventilator mode, duration of mechanical ventilation, and length of intensive care unit (ICU) stay in patients undergoing mechanical ventilation.

## Methods

### Study design

This two-center prospective observational study was conducted in the mixed medical/surgical ICUs of Tokushima University Hospital and Tokushima Prefectural Central Hospital between June 2016 and June 2018 (Additional file 1: Table S1). This study was approved by both clinical research ethics committees at Tokushima University Hospital (approval number 2593) and Tokushima Prefectural Central Hospital (approval number 1739). This trial was registered on a clinical trial (UMIN-Clinical Trials Registry: 000031316). At the time of enrollment, written informed consent was obtained from patients or their authorized surrogate decision makers.

### Study population

Consecutive adult patients who were expected to be mechanically ventilated longer than 48 h were enrolled in this study. Patients were recruited prospectively within 24 h following ICU admission. Patients who met the following criteria were excluded: age under 18 years, trauma or chest tube at the measurement point, diagnosis of primary neuromuscular disease.

### Measurements of diaphragm and intercostal muscle thickness

Imaging was performed with B-mode ultrasound using liner transducers. Diaphragm and intercostal muscle thickness were evaluated with serial ultrasound measurements on days 1, 3, 5, and 7. Recordings were discontinued at extubation, discharge from the ICU, or death of the patient, whichever occurs first.

All measurements were performed with patients in supine anatomical position, reclining in bed at a 30-degree angle. The transducer was perpendicularly placed on the right chest wall at the zones of apposition: between the eighth and tenth intercostal spaces, between the antero-axillary and the midaxillary lines, and 0.5–2 cm below the costophrenic sinus, as previously reported (Additional file 1: Figure S1) [6]. A mark was drawn on the patient’s skin to ensure consistency. Diaphragm and intercostal muscle thickness were measured three times at end expiration, and the mean value was recorded for evaluation. In this area, the diaphragm is observed as a three-layered structure, with the hypoechogenic muscular layer bordered by echogenic layers—the peritoneum and the diaphragmatic pleurae (Additional file 1: Figure S2). Thickening fraction of the diaphragm was calculated as [thickness at inspiration − thickness at expiration]/ [thickness at expiration] × 100 [7]. As intercostal muscles comprise internal and external intercostal muscle fibers that are difficult to discriminate by ultrasonography, intercostal muscles including the internal and external intercostal muscles were measured at the zone of apposition. In order to blind the data analysis from the patient status, the image was stored in ultrasound machine at the bedside and then, the same investigator measured the muscle thickness which was blinded from patients’ name and measurement days. Before commencing the study, intra- and inter-observer reproducibility were 0.92 and 0.96 for the diaphragm and 0.92 and 0.90 for the intercostal muscles, as assessed by two ICU physicians (Additional file 1: Figures S3-S6).

### Definitions

The study population was categorized into decreased thickness, increased thickness, and unchanged group, respectively in diaphragm and intercostal muscles. These categories were based on the change in muscle thickness over the study period, based on previous studies [1, 5, 8]. The decreased thickness group was defined as those exhibiting more than 10% decrease in thickness from day one to the lowest value over the measurement period. The increased thickness group was defined as more than 10% increase in thickness without more than 10% decrease, and the remaining patients were categorized into the unchanged group.

### Outcomes

The primary outcome was direction and rate of changes in the diaphragm and intercostal muscle thickness. The thickness change was defined as percent variation in muscle thickness compared with the admission day. The secondary outcomes were the relationship of changes in muscle thickness with patient characteristics; ventilator mode; duration of mechanical ventilation; length of ICU and hospital stay; reintubation and tracheostomy; the use of high-flow nasal cannula (HFNC) and noninvasive positive pressure ventilation (NPPV) after extubation; and medications including analgesics, sedatives, catecholamines, steroids, and muscle relaxants.

### Statistical analysis

No articles reporting on intercostal muscle atrophy in the ICU were found by the literature search. Therefore, a feasible sample size of 80 patients was planned for enrollment based on two studies on diaphragm atrophy (the average of 54 and 107 patients in these studies) [5, 8]. Continuous data were presented as means ± standard deviation or medians (interquartile range (IQR)), whereas categorical data were expressed as numbers (%). Changes in muscle thickness over time were assessed using a linear mixed model for repeated measures: statistical significance of changes in muscle thickness at each time point was also tested using 95% confidence intervals (CIs), with intervals not including zero considered as statistically significant. Kappa statistics was used to evaluate the relationship between changes in diaphragm and intercostal muscle thickness. Variables were compared using one-way analysis of variance or the Kruskal-Wallis test. Post hoc correction for multiple comparisons was performed with Dunnett’s or Steel’s test to compare decreased or increased thickness group versus unchanged group. Continuous outcomes were evaluated using Cox regression analysis, adjusted for age, sex, and APACHE II score. We presented cumulative incidence of liberation from mechanical ventilation, in which death was treated as a competing risk. Data were compared using Gray’s test with Bonferroni correction for two pairwise comparisons (significant at *p* < 0.025 vs. unchanged group). Data analyses were conducted using JMP 13.1.0 and SAS 9.4 (SAS Institute, Cary, NC). All statistical tests were two-tailed and a *p* value < 0.05 was regarded as statistically significant.

## Results

### Patient characteristics

The patient characteristics are shown in Table 1. Among a total of 84 patients, 80, 49, and 32 patients remained in the study on days 3, 5, and 7, respectively. Four patients were excluded due to only one measurement, leaving 80 patients in analysis. The mean age was 68 ± 14 years, and 54 patients were male. The median Acute Physiology and Chronic Health Evaluation II score was 24 (IQR, 19–30). The causes for admission were respiratory failure (28%), post-cardiac surgery (19%), and heart failure (10%).

**Table 1:** Patient characteristics

Characteristics	Overall (*n* = 80)
Age, mean ±SD, year	68 ± 14
Male/female	54/26
Body mass index, mean ± SD, kg/m^2^	24 ± 4
APACHE II score	24 (19–30)
SOFA, mean in the first 3 days	9 (5–12)
Sepsis (sepsis-3 criteria), *n* (%)	31 (39)
ICU admission reasons, *n* (%)	
Respiratory failure	22 (28)
Post-cardiac surgery	15 (19)
Heart failure	8 (10)
Sepsis, nonrespiratory	7 (9)
Stroke	7 (9)
Cardiac arrest	5 (6)
Traumas	2 (3)
Others	14 (18)

Data were expressed as median (IQR) unless otherwise indicated

*SD* standard deviation, *APACHE* Acute Physiology and Chronic Health Evaluation, *SOFA* Sequential Organ Failure Assessment

### Main outcomes

**Fig. 1:**
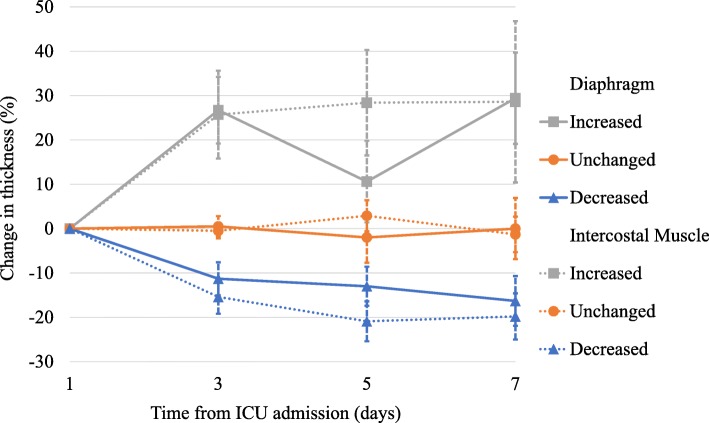
Time course of the diaphragm and intercostal muscle thickness. Time course for the measurement of the diaphragm and intercostal muscle thickness over the first 7 days of mechanical ventilation. The horizontal line represents the time from admission to the intensive care unit (ICU), and the vertical line represents the change in diaphragm and intercostal muscle thickness. Solid lines represent the changes in diaphragm muscle thickness, and dotted lines represent the changes in intercostal muscle thickness. Data are expressed as means and 95% confidence intervals

The diaphragm muscle thickness decreased, increased, and remained unchanged in 50 (63%), 15 (19%), and 15 (19%) patients, respectively. In the decreased thickness group, it decreased by 11.3% (95% CI, 7.6–15.0%), 13.0% (95% CI, 8.7–17.4%), and 16.3% (95% CI, 10.7–21.9%) on days 3, 5, and 7 (*p* < 0.01; Fig. 1). In the increased thickness group, it increased by 26.7% (95% CI, 19.1–34.2%), 10.6% (95% CI, 1.4–19.8%), and 29.4% (95% CI, 19.2–39.7%), on days 3, 5, and 7 (*p* < 0.01). Finally, in the unchanged group, it changed by 0.5% (95% CI, − 1.7–2.8%), − 2.0% (95% CI, − 7.7–3.7%), and − 0.02% (95% CI, − 6.9–6.9%) on days 3, 5, and 7 (*p* = 0.86).

Throughout the study period, the intercostal muscle thickness decreased, increased, and remained unchanged in 48 (60%), 15 (19%), and 17 (21%) patients, respectively. In the decreased thickness group, it decreased by 15.4% (95% CI, 11.5–19.2%), 20.9% (95% CI, 16.5–25.4%), and 19.8% (95% CI, 14.6–25.0%) on days 3, 5, and 7 (*p* < 0.01). In the increased thickness group, it increased by 25.7% (95% CI, 15.8–35.6%), 28.4% (95% CI, 16.5–40.3%), and 28.6% (95% CI, 10.4–46.8%) on days 3, 5, and 7 (*p* < 0.01). Finally, in the unchanged group, it changed by 0.5% (95% CI, − 2.2–1.2%), 2.9% (95% CI, − 0.5–6.4%), and − 1.3% (95% CI, − 5.2–2.7%) on days 3, 5, and 7 (*p* = 0.26).

### The relationship between the diaphragm and the intercostal muscle

**Fig. 2:**
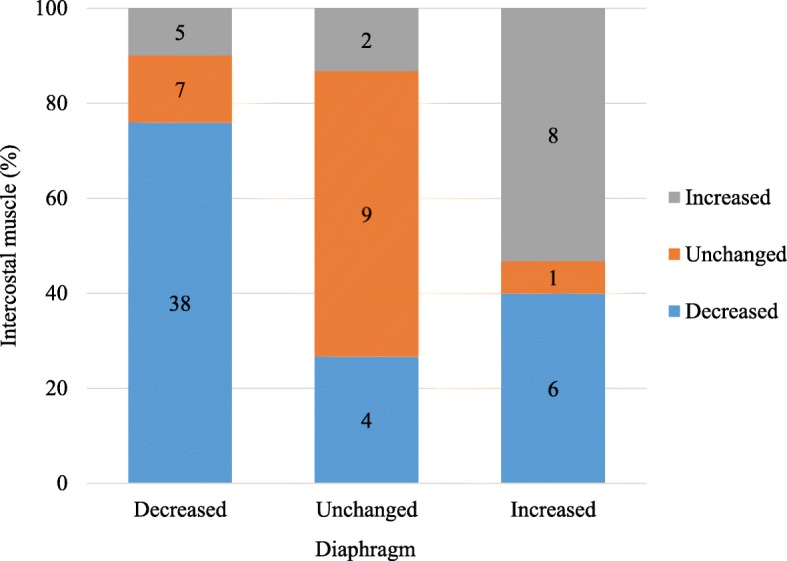
The relationship of changes in muscle thickness between the diaphragm and the intercostal muscles. Among groups stratified according to changes in diaphragm thickness, the percentages of patients with decreased, increased, or unchanged intercostal muscle thickness are shown by a bar graph. Numbers indicate the number of patients in each group. Changes in diaphragm and intercostal muscles thickness were associated with a kappa value 0.28 (95% confidence interval, 0.14–0.41, *p* < 0.001), suggesting a poor association

Out of fifty patients with the decreased diaphragm thickness, 38 patients (76%) exhibited decreased intercostal muscle thickness (Fig. 2). In the diaphragm unchanged group, 9 patients (60%) had no change in intercostal muscle thickness, whereas 8 patients (53%) in the increased diaphragm thickness group also had increased intercostal muscle thickness. In total, 55 patients (69%) exhibited muscle thickness changes in the same direction for both the diaphragm and intercostal muscle (decreased in 38 patients (48%), unchanged in 9 patients (11%), increased in 8 patients (10%)). The change in diaphragm thickness was associated with the change in intercostal muscle thickness, with the kappa value of 0.28 (95% CI, 0.14–0.41, *p* < 0.001), suggesting a poor association.

### The relationship of changes in muscle thickness with ventilator mode

Over the first 3 days, 75 patients (94%) were in assist-control ventilation mode (ACV), and all patients were controlled by pressure-control ventilation (Table 2). The thickening fraction of diaphragm was 7.1% (IQR, 4.3–13.1%) in decreased thickness, 8.7% (IQR, 5.8–11.9%) in increased thickness, and 8.7% (IQR, 6.5–16.1%) in unchanged (not in Table 2). There were no significant associations between thickening fraction and changes in diaphragm thickness (*p* = 0.65). Set inspiratory pressure above positive end-expiratory pressure was higher in the decreased and increased intercostal muscle thickness groups than the unchanged group (*p* = 0.02), but the inspiratory pressure was not significantly associated with the changes in diaphragm thickness (*p* = 0.28).

**Table 2:** The relationship of changes in diaphragm and intercostal muscle thickness with patient characteristics, medications, and ventilator mode

Variables	Change in diaphragm thickness				Change in intercostal muscle thickness			
	Decreased thickness (*n* = 50)	Unchanged (*n* = 15)	Increased thickness (*n* = 15)	*p* value	Decreased thickness (*n* = 48)	Unchanged (*n* = 17)	Increased thickness (*n* = 15)	*p* value
Age, mean ± SD, year	69 ± 2	69 ± 3	61 ± 3	0.10	70 ± 2	64 ± 3	66 ± 3	0.32
Male/female	34/16	10/5	10/5	0.99	32/16	13/4	9/6	0.60
Body mass index, mean ± SD, kg/m^2^	23 ± 1	24 ± 1	24 ± 1	0.72	23 ± 1^a^	26 ± 1	24 ± 1	< 0.01
APACHE II score	23 (18–29)	22 (20–31)	26 (22–32)	0.62	25 (17–30)	22 (17–27)	26 (22–32)	0.35
SOFA, mean in the first 3 days	9 (6–12)	8 (4–9)	10 (5–14)	0.33	9 (5–12)	7 (5–9)	9 (6–14)	0.22
Sepsis (sepsis-3 criteria), *n* (%)	22 (44)	5 (33)	4 (27)	0.43	18 (38)	6 (35)	7 (47)	0.77
Surgical admissions, *n* (%)	19 (38)	3 (20)	6 (40)	0.40	18 (38)	3 (18)	7 (47)	0.19
Medications, *n* (%)								
Catecholamine*	35 (70)	11 (73)	10 (67)	0.92	35 (73)	10 (59)	11 (73)	0.53
Neuromuscular blocking agents†	7 (14)	1 (7)	0 (0)	0.25	6 (13)	1 (6)	1 (7)	0.66
Steroids‡	18 (36)	5 (33)	2 (13)	0.25	18 (38)	3 (18)	4 (27)	0.29
Aminoglycoside	1 (2)	1 (7)	0 (0)	0.47	1 (2)	0 (0)	1 (7)	0.46
Opioid	44 (88)	13 (87)	11 (73)	0.37	42 (88)	16 (94)	10 (67)	0.07
Midazolam	18 (36)	7 (46)	7 (47)	0.64	20 (42)	7 (41)	5 (33)	0.84
Dexmedetomidine	24 (48)	6 (40)	4 (27)	0.33	23 (48)	4 (24)	7 (47)	0.20
Propofol	8 (16)	3 (20)	6 (40)	0.14	7 (15)	7 (41)	3 (20)	0.07
Ventilatory settings during first 3 days								
Controlled (ACV)/partial assist (PSV)	47/3	14/1	14/1	0.96	46/2	15/2	14/1	0.76
Set inspiratory pressure above PEEP	12 (10–12)	10 (10–12)	12 (10–14)	0.28	12 (10–13)^a^	10 (10–12)	12 (10–14)^a^	0.02
PEEP, cmH_2_O	8 (6–10)	8 (6–10)	6 (6–8)	0.31	8 (6–10)	8 (7–11)	8 (6–8)	0.11
Tidal volume/PBW, mL/kg	8.2 (7.3–9.6)	8.6 (7.6–10.2)	7.8 (7.2–9.7)	0.52	8.2 (7.2–9.7)	8.0 (7.8–9.7)	8.1 (7.2–9.7)	0.90

Data were expressed as median (IQR) unless otherwise indicated. *p* values were obtained using one-way analysis of variance (ANOVA) or the Kruskal-Wallis test

*SD* standard deviation, *APACHE* Acute Physiology and Chronic Health Evaluation, *SOFA* Sequential Organ Failure Assessment, *ACV* assist-control ventilation, *PSV* pressure-support ventilation, *PEEP* positive end-expiratory pressure, *PBW* predicted body weight

^a^Significant at *p* < 0.05 vs. Unchanged by post hoc Dunnett’s or Steel’s test

*Catecholamine (dopamine, dobutamine, noradrenaline, or adrenaline)

†Neuromuscular blockers with continuous use

‡Corticosteroids with intravenous or peroral use

### Patient outcomes

**Fig. 3:**
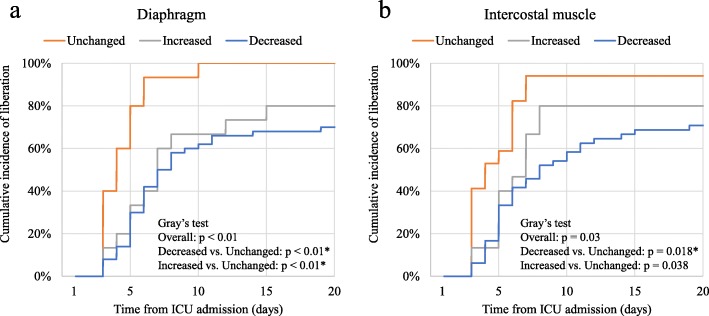
Cumulative incidence of liberation from mechanical ventilation by diaphragm or intercostal muscle thickness changes during the first week. Data were compared using Gray’s test with Bonferroni correction for two pairwise comparisons (significant at *p* < 0.025 vs. unchanged group). Death was treated as a competing risk. The horizontal line represents the time from admission to the intensive care unit (ICU), and the vertical line represents the cumulative incidence of liberation from mechanical ventilation. **a** Diaphragm: compared with unchanged, those with decreased or increased diaphragm thickness had a lower cumulative incidence of liberation from mechanical ventilation (*p* < 0.01 in both group). **b** Intercostal muscle: compared with unchanged, those with decreased diaphragm thickness had a lower cumulative incidence of liberation from mechanical ventilation (*p* = 0.018). VS, versus. *Significant at < 0.025 vs. unchanged group by Bonferroni correction

The duration of mechanical ventilation and the length of ICU stay were different among three groups (*p* < 0.01; Table 3). The duration of mechanical ventilation was longer in decreased diaphragm and intercostal muscle thickness group (median difference (MD), 3 days; 95% CI, 1–7 and MD, 3 days; 95% CI, 1–7, respectively). Moreover, it was longer in the increased diaphragm thickness group (MD, 2 days; 95% CI, 0–5, *p* = 0.04). Similarly, the length of ICU stay was longer in the decreased diaphragm and intercostal muscle thickness group (MD, 3 days; 95% CI, 1–7 and MD, 3 days; 95% CI, 1–7). In Cox regression analysis, the duration of mechanical ventilation and the length of ICU stay were also associated with decreased diaphragm and intercostal muscle thickness with the comparison to unchanged (*p* < 0.01; Table 4). Moreover, the duration of mechanical ventilation was associated with increased diaphragm thickness with the comparison to unchanged (*p* = 0.03). Compared with unchanged, those with decreased or increased diaphragm thickness during the first week had a lower cumulative incidence of liberation from mechanical ventilation (*p* < 0.01 in both group; Fig. 3). On the other hand, those with decreased intercostal muscle thickness had a lower cumulative incidence of liberation from mechanical ventilation (*p* = 0.018), and increased intercostal muscle thickness did not have the significant difference to unchanged (*p* = 0.038, significant level at *p* < 0.025 by Bonferroni correction).

**Table 3:** Outcomes

Outcomes	Change in diaphragm thickness				Change in intercostal muscle thickness			
	Decreased thickness (*n* = 50)	Unchanged (*n* = 15)	Increased thickness (*n* = 15)	*p* value	Decreased thickness (*n* = 48)	Unchanged (*n* = 17)	Increased thickness (*n* = 15)	*p* value
Duration of mechanical ventilation, day	7 (5–15)^a^	4 (3–5)	7 (4–12)^a^	< 0.01	8 (5–17)^a^	4 (3–6)	6 (5–8)	< 0.01
Length of ICU stay, day	10 (6–16)^a^	5 (5–7)	8 (5–15)	< 0.01	10 (6–18)^a^	5 (4–9)	7 (5–10)	< 0.01
Length of hospital stay, day	34 (22–54)	32 (13–118)	27 (11–65)	0.71	34 (22–72)	29 (13–46)	34 (16–65)	0.37
Reintubation, *n* (%)	9 (18)	1 (7)	1 (7)	0.36	10 (21)	1 (6)	0 (0)	0.07
Tracheostomy, *n* (%)	12 (24)	1 (7)	2 (13)	0.27	12 (25)	1 (6)	2 (13)	0.19
The use of HFNC, *n* (%)	29 (58)	8 (53)	7 (47)	0.73	27 (56)	9 (53)	8 (53)	0.96
The use of NPPV, *n* (%)	3 (6)	0 (0)	0 (0)	0.39	2 (4)	1 (6)	0 (0)	0.66
Mortality in the ICU, *n* (%)	10 (20)	0 (0)	1 (7)	0.10	8 (17)	0 (0)	3 (20)	0.17
Mortality in the hospital, *n* (%)	18 (36)	1 (7)	5 (33)	0.09	17 (35)	1 (6)	6 (40)	0.048

Data were expressed as median (IQR) unless otherwise indicated. *p* values were obtained using one-way analysis of variance (ANOVA) or the Kruskal-Wallis test

*ICU* intensive care unit, *HFNC* high-flow nasal cannula, *NPPV* noninvasive positive pressure ventilation

^a^Significant at *p* < 0.05 vs. unchanged by post hoc Dunnett’s or Steel’s test

**Table 4:** Outcomes by multivariate analysis

Outcomes	Change in diaphragm thickness		Change in intercostal muscle thickness	
	Decreased thickness vs. unchanged	Increased thickness vs. unchanged	Decreased thickness vs. unchanged	Increased thickness vs. unchanged
Duration of mechanical ventilation, day	4.19 (2.14–7.93)*	2.38 (1.08–5.29)†	2.87 (1.53–5.21)*	1.71 (0.79–3.81)
Length of ICU stay, day	3.44 (1.77–6.45)*	1.99 (0.92–4.39)	2.58 (1.39–4.63)*	1.43 (0.66–3.16)
Length of hospital stay, day	1.34 (0.66–2.60)	0.89 (0.36–2.28)	2.04 (1.06–3.81)†	2.21 (0.94–5.61)

Data are expressed as hazard ratio (95% confidence interval) with intervals not including zero considered as statistically significant. Cox regression analysis was used for the analysis, adjusted for age, sex, and APACHE II score

*ICU* intensive care unit

*p < 0.01

†*p =* 0.03

## Discussion

This prospective observational ultrasonography study investigated the time course of change in diaphragm and intercostal muscle thickness daily for up to 7 days in mechanically ventilated patients. The diaphragm muscle thickness can decrease, increase, or remain unchanged during mechanical ventilation. A similar change was observed in intercostal muscles, which are also essential respiratory muscles. Our study found that atrophy of diaphragm or intercostal muscles was associated with prolonged mechanical ventilation and length of ICU stay.

While the diaphragm is the main respiratory muscle, accounting for 60–80% of the inspiratory work [9], other respiratory muscles also play an important role in moving the rib cage, especially in cases where the diaphragm function deteriorates or work associated with breathing increases [3]. Several studies detected rib cage muscle activity at high inspiratory efforts by electromyography [10, 11]. In one study, the activity of rib cage muscles exceeded that of the diaphragm during increased inspiratory workload [9]. Rib cage muscles comprise intercostal, sternocleidomastoid, scalene, pectoralis, serratus, oblique, transverse abdominis, and rectus abdominis muscles. Among these, the intercostal muscles are the most important respiratory muscles that significantly contribute to the movement of the rib cage [9]. In patients with chronic obstructive pulmonary disease, intercostal muscle atrophy occurs in advanced stages [12], and the decreased mass of intercostal muscles is associated with exacerbation [13]. Our findings highlight the important role of intercostal muscles in critically ill patients and suggest a possible association of their atrophy with difficult weaning from mechanical ventilation and prolonged ICU stay.

Several studies reported on rib cage muscle atrophy under mechanical ventilation. In one study, mechanical ventilation led to intercostal muscle atrophy as well as diaphragm muscle in animals [2]. Capdevila et al. suggested that 48 h of mechanical ventilation led to a 29% decrease in intercostal muscle mass in rabbits [2]. Another study by Levine et al. investigating atrophy of the pectoralis major muscle and the diaphragm in mechanically ventilated patients [14] found that the muscle fibers of the pectoralis major muscle did not decrease, although the cross-sectional area of the diaphragm muscle fibers was decreased. However, that study compared a limited number of 22 patients with different pathologies. In contrast, Nakanishi et al. reported that biceps brachii, which can also function to expand rib cage, showed significant progressive atrophy in critically ill patients [15]. Thus, rib cage muscles can undergo atrophy in critically ill patients.

In contrast to previous studies reporting the incidence of diaphragm atrophy at 41–44% [1, 5], 63% of the patients in the current study had decreased diaphragm thickness, which we propose might be due to two potential explanations. First, majority of the patients in the current study were controlled by ACV, which was previously shown to be associated with excessive respiratory support and neglected spontaneous breathing [16, 17]. Therefore, an average thickening fraction was 8% in the current study, which should be approximately 25% in individuals with normal breathings. Second, the mean patient age was higher in the current study compared with previous studies (68 vs. 59–60 years), which is associated with prominent muscle atrophy and decreased functional status [18]. As previously reported, we also found that decreased diaphragm thickness was associated with the duration of mechanical ventilation and the length of ICU stay, and diaphragm thickness decreased quickly in the early phase after mechanical ventilation [1, 8].

The increased diaphragm thickness was observed in 19% of the patients and remained unchanged in another 19%, which were previously reported as 12–24% and 35–44%, respectively [1, 5]. Increased diaphragm thickness was associated with prolonged mechanical ventilation. Increased diaphragm thickness has been garnering increased interest based on a study by Goligher et al. who recently reported that increased diaphragm thickness predicted prolonged mechanical ventilation (OR, 1.38; 95% CI, 1.00–1.90). The rate of increased diaphragm thickness in the current study is comparable with those reported in previous studies [1, 5]. However, increased diaphragm thickness was not significantly associated with excessive thickening fraction as previously reported [5]. The potential reason is the limited number of high thickening fraction, defined as more than 25% thickening (2.5% in our study population vs. 12.3% in Goligher’s study) [1]. Despite the relatively lower thickening fraction in the current study, increased thickness was observed in 15 patients, suggesting increased diaphragm thickness is likely multifactorial. The clinical significance of increased diaphragm thickness is unclear in this research because of the relatively lower number of increased thickness in the study cohort (15 with increased thickness vs. 50 patients with atrophy). Furthermore, most studies on diaphragm focuses on atrophy and not increased thickness [8, 17, 19, 20], reflected on the paucity of data on its increased thickness [1, 5]. Increased diaphragm thickness needs further investigation.

The changes in the diaphragm and intercostal muscle thickness showed a poor association (κ = 0.27, *p* < 0.01). Although the association was poor, the atrophy of both the diaphragm and intercostal muscles was related to prolonged mechanical ventilation. This result shows both of the diaphragm and intercostal muscles are important for critically ill patients, but different factors may influence the atrophy. The extent of disuse may be different because diaphragm and rib cage muscles contribute to respiration differently [9]. In spontaneous breathing patients, mechanical ventilation can support some part of diaphragm function and may disuse intercostal muscles. On the other hand, intercostal muscles may exceed the diaphragm function in increased respiratory workload [3]. Therefore, the relationship was limited to a poor association between the diaphragm and the intercostal muscles.

Ventilator settings caused contradictory results in diaphragm and intercostal muscle thickness. We did not find a significant difference in the inspiratory pressure between the patients with changed and preserved diaphragm thickness. The limited range of set inspiratory pressure (IQR, 12–14 cmH_2_O) may explain our results, since a previous study compared patients with a set inspiratory pressure of ≥ 12 cmH_2_O to those with a set inspiratory pressure of 5–12 cmH_2_O [20]. Conversely, high inspiratory pressure was associated with both the decreased and increased thickness of intercostal muscles. Changes in thickness were easier to monitor for intercostal muscles than the diaphragm because of the bigger muscle size (2.1 mm for diaphragm vs. 4.2 mm for intercostal muscles). Therefore, the impact of pressure was likely detected in the intercostal muscles. As excessive inspiratory support causes atrophy of the disused muscle, excessive pressure might cause injury to respiratory muscles and structural changes with gradual increased thickness. Since set inspiratory pressure does not reflect the actual pressure loaded to respiratory muscles, transpulmonary pressure should be measured to elucidate its precise influence. Similarly, the significance of the observed association between low body mass index (BMI) and intercostal muscle atrophy is unclear. It is possible that this result reflects the association of low BMI with malnutrition and increased respiratory workload. Further elucidation of this aspect is limited due to the small study sample size that was not powered to detect the significant differences.

Changes in respiratory muscle thickness occur frequently in critically ill patients. As reported for the diaphragm, intercostal muscles exhibit significant changes in these patients, and the underlying causes are multifactorial. The severity of the clinical status, medications, and ventilatory settings should be further investigated as potential factors. Compared with immobilized limb muscles, respiratory muscles are permanently activated by the ventilator [15]. Therefore, the duration and settings of mechanical ventilation may be the key factors to preserve respiratory muscles. Whether prolonged mechanical ventilation led to respiratory muscle atrophy or respiratory muscle atrophy induced prolonged mechanical ventilation remains to be elucidated. Weaning patients from mechanical ventilation may be essential to prevent or minimize respiratory muscle changes. It is suggested that maintaining and improving respiratory muscles thickness is essential in order to do fast-track extubation in the ICU. Further studies are needed to determine approaches to maintain respiratory muscles. Taken together, the current findings emphasize that it is essential to monitor changes in diaphragm and intercostal muscles.

## Limitations

The current study has several limitations. First, although the patients were enrolled from two ICUs, this cohort study was limited to one country. Second, ultrasound evaluation of intercostal muscles is not established well, while reproducibility of measurement was good. Third, investigators performing measurements were not blinded to patients’ condition, although image analyses were blinded. Fourth, we classified decreased thickness first, and then, we classified increased thickness. Therefore, this classification is not fair to increased thickness. Finally, changes in intercostal muscle thickness may depend on the specific intercostal muscle that was measured.

## Conclusion

This two-center prospective observational study evaluating changes in diaphragm and intercostal muscle thickness in mechanically ventilated patients measured with ultrasound revealed that decreased diaphragm and intercostal muscle thickness were frequently seen in patients under mechanical ventilation. The decreased diaphragm and intercostal muscle thickness were associated with prolonged mechanical ventilation and length of ICU stay.

## Supplementary information

**Additional file 1: Table S1.** Facility and equipment in this two-center prospective observational study. **Figure S1.** Anatomical structures at the zone of apposition. **Figure S2.** Ultrasound image of the diaphragm and intercostal muscles from the intercostal view. **Figure S3.** Intra-observer reproducibility of diaphragm. **Figure S4.** Inter-observer reproducibility of diaphragm. **Figure S5.** Intra-observer reproducibility of intercostal muscle. **Figure S6.** Inter-observer reproducibility of intercostal muscle.

## Data Availability

The datasets used and/or analyzed during the current study are available from the corresponding author on reasonable request.

## Abbreviations

ACV: Assist-control ventilation
BMI: Body mass index
CI: Confidence interval
HFNC: High-flow nasal cannula
ICU: Intensive care unit
IQR: Interquartile range
NPPV: Noninvasive positive pressure ventilation
VS: Versus
