# Supplementary material for: Change in diaphragm and intercostal muscle thickness in mechanically ventilated patients: a prospective observational ultrasonography study
Source: J Intensive Care. 2019 Dec 2;7:56. doi: 10.1186/s40560-019-0410-4 (PMC6886193; doi:10.1186/s40560-019-0410-4)
Supplement: Supplementary file 1 — Additional file 1: Table S1. Facility and equipment in this two-center prospective observational study. Figure S1. Anatomical structures at the zone of apposition. Figure S2. Ultrasound image of the diaphragm and intercostal muscles from the intercostal view. Figure S3. Intra-observer reproducibility of diaphragm. Figure S4. Inter-observer reproducibility of diaphragm. Figure S5. Intra-observer reproducibility of intercostal muscle. Figure S6. Inter-observer reproducibility of intercostal muscle. [file 40560_2019_410_MOESM1_ESM.docx]

**Table S1. Facility and equipment in this two-center prospective observational study**

| **Facility and equipment** | **Tokushima university hospital** | **Tokushima prefectural central hospital** |
| --- | --- | --- |
| ICU type | Closed | Open |
| Admission type | mixed medical/surgical ICU | mixed medical/surgical ICU |
| Number of beds | 10 | 8 |
| Annual admissions | 400 to 500 | 400 to 500 |
| Ultrasounds | HI VISION Preirus, Hitachi Medical Corporation, Tokyo, Japan | LOGIQ P9, GE healthcare, WI, USA |
| Transducers | EUP-L73S liner transducer, Hitachi Medical Corporation, Tokyo, Japan | 12L-RS liner transducer, GE healthcare, WI, USA |

**Figure S1. Anatomical structures at the zone of apposition.**

The right side depicts the relationship between the lungs, rib cage, and diaphragm. The anatomical structures including ribs, intercostal muscles, and diaphragm are magnified on the left side. Intercostal muscle and diaphragm thickness were measured with ultrasound using a probe positioned perpendicularly on the chest wall.

**
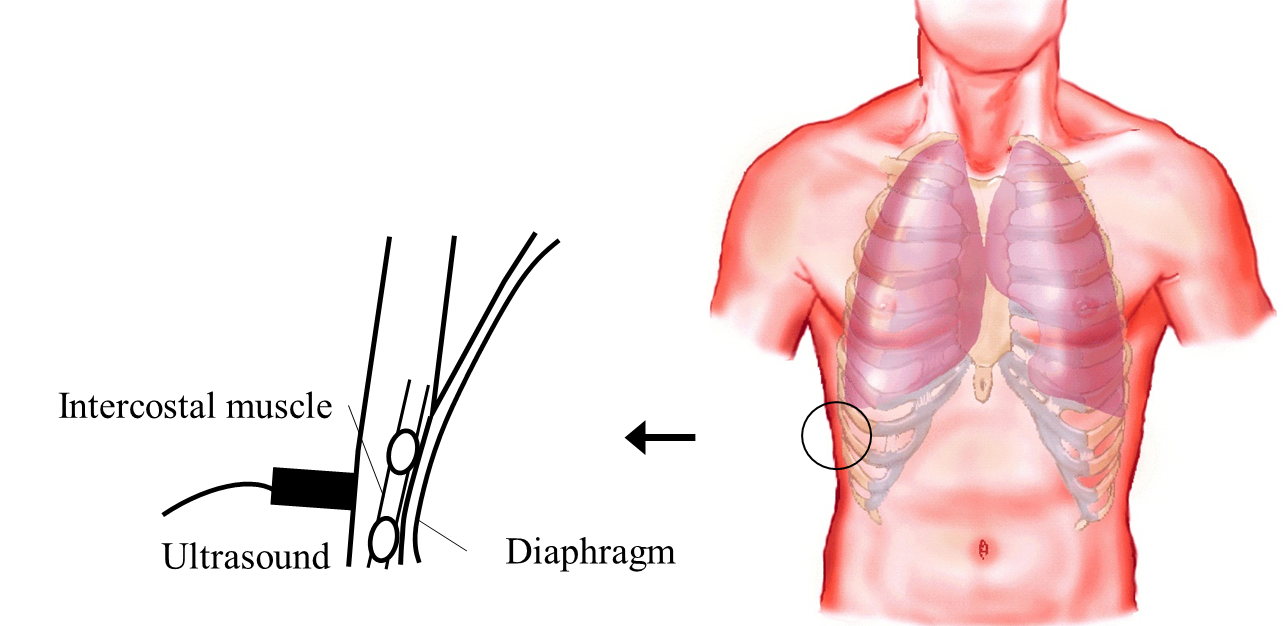
**

**Figure S2. Ultrasound image of the diaphragm and intercostal muscles from the intercostal view.**
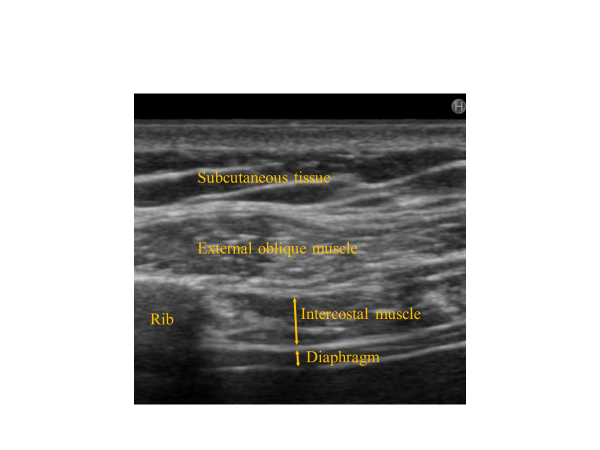


**Figure S3. Intra-observer reproducibility of diaphragm**

Reproducibility was assessed for 20 critically ill patients. Pearson’s correlation coefficient and Bland-Altman plot were determined by using JMP statistical software version 13.1.0 (SAS Institute Inc., Cary, NC). Abbreviation; LOA, Limit of agreement

LOA

LOA

R^2^=0.92, p<0.0001

|  | Estimated value | P Value |
| --- | --- | --- |
| y | 0.06 | p=0.65 |
| x | 0.99 | p<0.0001 |

| Bias | 0.045 ± 0.156 |
| --- | --- |
| 95% LOA | -0.26 to 0.35 |

**Figure S4. Inter-observer reproducibility of diaphragm**

LOA

LOA

R^2^=0.96, p<0.0001

|  | Estimated value | P Value |
| --- | --- | --- |
| y | -0.14 | p=0.19 |
| x | 1.13 | p<0.0001 |

| Bias | 0.090 ± 0.137 |
| --- | --- |
| 95% LOA | -0.18 to 0.36 |

**Figure S5. Intra-observer reproducibility of intercostal muscle**

LOA

LOA

R^2^=0.92, p<0.0001

|  | Estimated value | P Value |
| --- | --- | --- |
| y | 0.32 | p=0.31 |
| x | 0.92 | p<0.0001 |

| Bias | -0.025 ± 0.386 |
| --- | --- |
| 95% LOA | -0.78 to 0.73 |

**Figure S6. Inter-observer reproducibility of intercostal muscle**

LOA

LOA

R^2^=0.90, p<0.0001

|  | Estimated value | P Value |
| --- | --- | --- |
| y | 0.33 | p=0.34 |
| x | 0.93 | p<0.0001 |

| Bias | 0.005 ± 0.326 |
| --- | --- |
| 95% LOA | -0.63 to 0.64 |
